# Comparative Analysis of Tomato Brown Rugose Fruit Virus Isolates Shows Limited Genetic Diversity

**DOI:** 10.3390/v14122816

**Published:** 2022-12-17

**Authors:** Peter Abrahamian, Weili Cai, Schyler O. Nunziata, Kai-Shu Ling, Namrata Jaiswal, Vessela A. Mavrodieva, Yazmín Rivera, Mark K. Nakhla

**Affiliations:** 1 USDA-APHIS-PPQ Plant Pathogen Confirmatory Diagnostics Laboratory, Laurel, MD 20708, USA; 2 USDA-Agricultural Research Service, U.S. Vegetable Laboratory, Charleston, SC 29414, USA

**Keywords:** tobamovirus, polymorphism, population, emerging, United States, high throughput sequencing

## Abstract

Tomato is an important vegetable in the United States and around the world. Recently, tomato brown rugose fruit virus (ToBRFV), an emerging tobamovirus, has impacted tomato crops worldwide and can result in fruit loss. ToBRFV causes severe symptoms, such as mosaic, puckering, and necrotic lesions on leaves; other symptoms include brown rugose and marbling on fruits. More importantly, ToBRFV can overcome resistance in tomato cultivars carrying the *Tm-2^2^* locus. In this study, we recovered ToBRFV sequences from tomato seeds, leaves, and fruits from the U.S., Mexico, and Peru. Samples were pre-screened using a real-time RT-PCR assay prior to high-throughput sequencing. Virus draft genomes from 22 samples were assembled and analyzed against more than 120 publicly available genomes. Overall, most sequenced isolates were similar to each other and did not form a distinct population. Phylogenetic analysis revealed three clades within the ToBRFV population. Most of the isolates (95%) clustered in clade 3. Genetic analysis revealed differentiation between the three clades indicating minor divergence occurring. Overall, pairwise identity showed limited genetic diversity among the isolates in this study with worldwide isolates, with a pairwise identity ranging from 99.36% and 99.97%. The overall population is undergoing high gene flow and population expansion with strong negative selection pressure at all ToBRFV genes. Based on the results of this study, it is likely that the limited ToBRFV diversity is associated with the rapid movement and eradication of ToBRFV-infected material between countries.

## 1. Introduction

Tomato brown rugose fruit virus is a member of the genus Tobamovirus and family Virgaviridae [1]. Tomato brown rugose fruit virus (ToBRFV) is a recently characterized virus known to infect tomatoes and pepper in the field [1,2]. Symptoms of ToBRFV are characterized by mild to severe mosaic on leaves, necrosis, and puckering on leaves, whereas fruits show marbling and brown rugose [1,2]. ToBRFV infects tomatoes and peppers in the field [1,2]. In addition, host range studies under laboratory conditions showed that ToBRFV can infect several solanaceous plants, such as petunias, tobacco, and weeds, such as black nightshade and *Chenopodium* spp., inciting mild or no symptoms [2,3]. ToBRFV was initially observed in Jordan and Israel simultaneously [1,2]. Like other tobamoviruses, ToBRFV is efficiently transmitted through mechanical inoculation [1,2]. Furthermore, ToBRFV spreads through the movement of infected seeds and seedlings [4], direct plant-to-plant contact, and direct human contact [5]. Bumble bee species, such as *Bombus terrestris*, can also transmit the virus in the field [6]. ToBRFV is mainly found on the seed coat but not in the embryo and can reach a seed contamination rate of 100% from infected fruits [4,7]. Transmission of the virus from seed to seedling occurs upon germination caused by the contaminated seed coat with a transmission rate from as low as 0.08% and up to 2.8% [4,7]. This virus has garnered much attention due to its ability to infect tomato plants carrying the *Tm-2^2^* locus, a gene conferring strong resistance against tobacco mosaic virus (TMV) and tomato mosaic virus (ToMV) and near complete resistance against tomato mottle mosaic virus (ToMMV) [2,3]. Therefore, ToBRFV is considered a highly damaging virus [2,8,9,10].

To date, ToBRFV has been reported from Germany [11], Turkey [12], Italy (Sicily) [13], the United States (California, [14]; Florida [15]), the United Kingdom [16], China [17], Palestine [18], Canada [19], Mexico [20], Greece [21], Egypt [22], Spain [23], the Netherlands [24], Cyprus [25], Czech Republic [25], France [25], Hungary [25], Malta [25], Poland [25], Switzerland [25], Belgium [25], Norway [25], Hungary [25], Bulgaria [25], Austria [25], Slovenia [25], Estonia [25], Syria [26], Portugal [25], Iran [27], and Saudi Arabia [28]. Several of the above-mentioned countries have swiftly eradicated the disease from tomato-growing areas with limited presence, while others have only detected ToBRFV in imported plant materials [25]. Nevertheless, incidence reports of ToBRFV continue to be rapidly increasing worldwide.

The genome of ToBRFV is 6,392 nt long and has four open reading frames, 183 kDa and 126 kDa replication proteins, a movement protein (MP), a coat protein (CP), and 5′ and 3′ untranslated regions [2]. The genetic diversity of ToBRFV is not well studied, and so far, only a limited number of studies have examined the diversity in the ToBRFV population. Most sequenced ToBRFV genomes originated from isolates recovered in the Netherlands [24,29]. Moreover, van de Vossenberg et al. [24] developed a NextStrain database to track ToBRFV outbreaks in the Netherlands and sequenced two ToBRFV population sets, each containing 54 and 47 genomes. Genetic diversity was low between the sequenced isolates in both populations, and Dutch isolates clustered into three groups in the first dataset [24]. In the second NextStrain build, a divergent isolate recovered from seeds originating from Peru was found to represent a new genotype, and more variation is expected with more sequenced isolates [29]. A drawback of both studies is the strong bias towards Dutch isolates (82 out of 118 sequences), which affects the proper determination of transmission links [24,29]. Furthermore, virus genomes from other geographical areas, such as the American continent, are underrepresented in public databases. Comparative analysis studies based on the first available ToBRFV genomes were carried out to map the resistance-breaking mutation compared to similar tobamoviruses [30]. The MP gene was later identified and experimentally validated as the genetic determinant for overcoming *Tm-2^2^* resistance [8,10]. Further characterization revealed that the specific amino acid mutations of the following residues, H67, N125, K129, A134, I147, or I168, in the MP completely abolished infection of ToBRFV in *Tm-2^2^* plants [10]. By sequencing additional genomes of North American isolates and comparing those to publicly available genomes, the diversity of ToBRFV populations can be better understood. Effective screening of isolates and determining ToBRFV sequence diversity in circulating plant material is important for ensuring the integrity of deployed diagnostic assays.

In the U.S., a federal order was issued in 2019 by the U.S. Department of Agriculture to limit the introduction of ToBRFV into the country by screening tomato and pepper seed and plant materials. In this study, we recovered ToBRFV from suspected tomato plant materials from various locations over a time frame of four years. The objectives of this study were to: (1) sequence ToBRFV genomes using high-throughput sequencing, (2) characterize the genetic diversity of ToBRFV in the U.S. and worldwide, and (3) identify elements driving evolutionary changes in ToBRFV genomic analysis of isolates collected in North America.

## 2. Materials and Methods

### 2.1. Plant Material

Tomato seeds, leaves, and fruits were received from greenhouse outbreaks, seed detections, and retail stores and processed at the Plant Pathogen Confirmatory Diagnostics Laboratory (USDA-APHIS) and at the U.S. Vegetable Laboratory (USDA-ARS) (Table 1). Total RNA was extracted from symptomatic leaves and fruits using the Qiagen RNeasy Plant Mini Kit (Qiagen, Hilden, Germany) or from seeds using the Sbeadex maxi plant kit (LGC Genomics, Middlesex, United Kingdom). The quality of the total RNA was evaluated using the 4200 TapeStation System (Agilent Technologies, Santa Clara, CA, USA). Total RNA concentrations were measured using a Qubit Fluorometer (Invitrogen, Waltham, MA, USA).

### 2.2. Real-Time RT-PCR Assays

ToBRFV was detected in the samples using a real-time RT-PCR assay developed by Chanda et al. [3]. Briefly, the real-time RT-PCR targeted the ToBRFV movement protein (MP) using primers (ToBRFV-F1, 5′-GCCCATGGAACTATCAGAAGAA-3′; ToBRFV-R1, 5′-TTCCGGTCTTCGAACGAAAT-3′) and a TaqMan probe (ToBRFV-P1, FAM-AGTCCCGATGTCTGTAAGGCTTGC-TAMRA) [3]. The real-time RT-PCR assays were carried out in a QuantStudio^TM^ 5 (Applied Biosystems, Waltham, MA, USA).

### 2.3. Library Preparation and Sequencing

The Illumina TruSeq Stranded Total RNA kit with Ribo-Zero Plant (Illumina, San Diego, CA, USA) was used for constructing libraries according to the manufacturer’s instructions. Total RNA used in the real-time PCR assays was also used for library preparation. A total of 22 libraries were prepared. Libraries were tagged with unique dual indexes and pooled before loading for sequencing. Libraries were sequenced using a 1 × 75-bp Illumina single-end read v2.5 sequencing kit on an Illumina NextSeq 550 sequencer (Illumina, Inc., San Diego, CA, USA).

### 2.4. Bioinformatics Analyses

An in-house bioinformatics pipeline was developed for the detection of viruses in the suspect samples. The pipeline workflow is as follows: reads were trimmed using Trimmomatic v. 0.39 [31], host reads were filtered out by mapping against the tomato genome (GCF_000188115.4) using bowtie using default settings, non-host reads were de novo assembled using Metaviral SPAdes v. 3.15.02 [32] using default settings, and viral contigs were analyzed using BLASTn using a cutoff value of 0.001 against an in-house curated viral database generated from GenBank plant virus and viroid sequences. Non-host reads were also re-mapped against the ToBRFV reference genome, isolate Tom1-Jo (NC_028478.1), using the Geneious RNA mapper with a 7% maximum mismatch per read in Geneious v. 2020.0.5. Non-host reads were also mapped against the assembled contigs to verify single nucleotide polymorphisms (SNPs) compared to the reference genome. The final contigs consisted of near-complete genomes with a few missing nucleotides in the 5′ or 3′ untranslated regions (UTR).

### 2.5. Phylogenetic Analysis

The sequences of available ToBRFV genomes (*n* = 123) were retrieved from GenBank and used in this study for comparative analysis. The genomes included isolates recovered from outbreak hotspots and different localities. A multiple sequence alignment of all publicly available ToBRFV genomes and those obtained in this work (*n* = 22) was performed using MAFFT v. 7.450 in Geneious Prime v. 2020.0.5. The 5′ and 3′ UTRs were trimmed from all aligned genomes prior to phylogenetic analysis. Furthermore, a multiple sequence alignment was conducted on each of the four ToBRFV genes, 183 kDa, 126 kDa, MP, and CP. The best-fit model was determined using MEGAX v. 10.2.2 and selected based on the lowest Akaike information criterion (AIC). The General time reversible (GTR) model was selected for the dataset containing the full-genome sequences, the 183 kDa gene, and 126 kDa gene. The HKY85 and TN93 were selected for the CP and MP sequence alignments. Phylogenetic analysis was conducted using PhyML v. 3.3 using default parameters and selection of the best-fit model. The tree was subject to 500 bootstraps. The tree was optimized for topology, length, and rate.

### 2.6. Estimation of Genetic Diversity Parameters and Neutrality Tests

Genetic diversity parameters for the U.S. isolates sequenced in this study and for all publicly available ToBRFV genomes were determined using DnaSP v6.12.03. The following criteria were calculated using DnaSP: number of segregating sites (S), nucleotide diversity (π), and haplotype diversity (h). A sliding window of 100 bp with a step size of 25 nt was selected for visualizing π across the genome. Tajima’s D, Fu and Li’s D * and F * tests were calculated to evaluate the null hypothesis of neutral evolution. Tajima’s D value of less than zero indicates population expansion after a recent bottleneck or abundance of rare polymorphisms. Whereas Tajima’s D values greater than zero indicate contraction in the population and the absence of rare polymorphisms. The ratio of the number of nonsynonymous substitutions to the number of synonymous substitutions (dN/dS) was also calculated as an indicator of selection pressure. A value of less than one indicates negative selection, and a value greater than one indicates positive selection of the mutation. Palestinian ToBRFV isolate (GenBank accession no. MK165457.1) was excluded from the RdRp genetic diversity parameter analysis due to a frameshift and early stop codon in the gene.

### 2.7. Estimation of Gene Flow and Genetic Differentiation

The degree of gene flow (Fst), i.e., the movement of genes in and out of the population, and the number of migrants per generation (Nm) were estimated based on the structure observed in the phylogenetic tree. Each of the three clades was assigned as a population and compared with each other. Fst is a good indicator of the overall divergence between populations. Fst ranges between 0 to 1 from low to highly structured populations, respectively. For viruses, frequent gene flow is considered to occur when Fst < 0.33 and Nm < 1 [33,34]. Moreover, Nm > 1 indicates no or little genetic drift, supporting gene flow, whereas Nm < 1 indicates strong genetic drift.

Moreover, to estimate genetic differentiation between and within the sub-populations of ToBRFV, independent nucleotide test statistics such as Kst * (values close to zero indicate no differentiation), Z * (logarithm of the Z-rank statistic), and the nearest neighbor statistic (Snn) were determined. Snn value close to one indicates two populations are highly differentiated. All differentiation tests were subject to a 1000-times permutation test, and *p*-values were determined in DnaSP. The null hypothesis of no genetic differentiation is rejected when the values of Kst *, Z *, and Snn are significant.

### 2.8. Sequence Submission

The ToBRFV near-complete genomes retrieved from the consensus sequence of the de novo and reference assemblies were submitted to the NCBI database. The accession numbers are OM892670-OM892691.

## 3. Results

### 3.1. High-Throughput Sequencing Data

**Figure 1:**
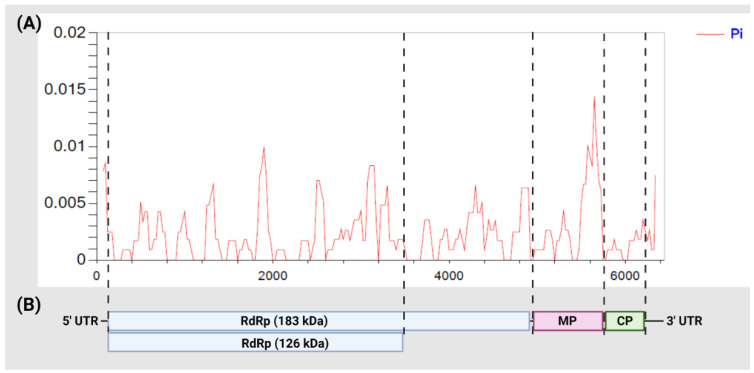
(**A**) Nucleotide diversity (*π*) distribution across all tomato brown rugose fruit virus (ToBRFV) isolates sequenced in this study. The *y*-axis and the *x*-axis represent *π* and the nucleotide position, respectively. (**B**) Genome organization of ToBRFV. RdRp: RNA-dependent RNA polymerase; MP: movement protein; CP: coat protein; UTR: untranslated region.

Tomato samples were tested prior to sequencing with a specific ToBRFV real-time RT-PCR assay and showed Ct values ranging between 4.9 and 24.8 (Table 1). HTS data showed that all samples produced reads with very high read depth for ToBRFV ranging from 78× and up to 773,145×. Two samples, S23 and S24, had a lower number of ToBRFV read coverage than the remaining samples. Both samples produced higher Ct values compared to the remaining sequenced samples. Nevertheless, all samples produced sufficient reads that mapped to the reference sequence with up to 100% genome coverage. The assembled ToBRFV contigs were near complete genomes with a minimum coverage of 99.4% (Table 2). All 22 ToBRFV genomes contain sequences for the RNA-dependent RNA polymerase subunits (183 kDa and 126 kDa), movement protein (30 kDa), and coat protein (17.5 kDa) (Figure 1).

### 3.2. Diversity among ToBRFV Isolates

All ToBRFV sequenced isolates in this study had the same length for all four open reading frames. Nucleotide identity among all sequenced isolates was lowest for the MP ORF, whereas the highest nucleotide identity was observed in the CP ORF (Table 3). On the other hand, the amino acid identity was highest for both the 126 kDa and 183 kDa ORFs at 99.96% and 99.94%, respectively, and the lowest amino acid identity was observed in the MP gene at 99.41%. The overall pairwise identity for the sequenced genomes ranged between 99.51% to 100%. Moreover, the number of SNPs across the full genome between any two isolates ranged from 0 to 30 nt. The CP ORF had the lowest number of mutations, of which four resulted in an amino acid change and one synonymous mutation (Table 3). The highest number of synonymous and non-synonymous mutations was observed in the 183 kDa ORF, and the MP ORF had the second-highest number of non-synonymous mutations (Table 3). The nucleotide diversity sliding window showed higher diversity across the RdRp region and the MP compared to the CP ORF (Figure 1).

ToBRFV U.S. isolates were compared to the reference sequence and other publicly available genomes. The U.S. isolates had an insertion in the 3′UTR, which is also present in the worldwide isolates, compared to the reference isolate Tom1-Jo from Jordan. No insertions or deletions were observed in any of the ORFs across all isolates except for the Palestinian isolate, which was excluded from some analyses. Pairwise comparison between isolates in this study and all other publicly available genomes showed a similarity between 99.36% and 99.97%. Furthermore, the sequenced isolates showed 2 to 39 nt differences with all other publicly available complete genome sequences. Previously sequenced U.S. isolate CA18-01 from California [35] showed a pairwise identity of 99.93% to 99.94%, with the four Californian isolates sequenced in this study.

### 3.3. Mutations Affecting Movement Protein–Tm-2^2^ Interaction

Six amino acid sites spanning the movement proteins were analyzed and compared to the recently reported critical mutations, H67, N125, K129, A134, I147, or I168, which can render ToBRFV non-infectious in tomatoes carrying the *Tm-2^2^* locus [10]. Across all 22 isolates that were sequenced, we found six amino acid mutations in the *MP* gene. A single critical mutation, A134T, was found in sample S20. The remaining five mutations are non-critical mutations and located in the C-terminus (186–266) portion of the gene, which is not essential for overcoming Tm-2^2^ resistance. Upon analysis of all 144 sequences, inclusive of the isolates sequenced here, a total of 19 amino acid mutations were identified spanning the *MP* gene, 11 of which are in the C-terminus. Five mutations were in the core region (60–186), previously mapped to be essential for ToBRFV pathogenicity, four of which were non-critical mutations, and only one A134T in S20 is a critical mutation.

### 3.4. Phylogenetic Analysis of ToBRFV

**Figure 2:**
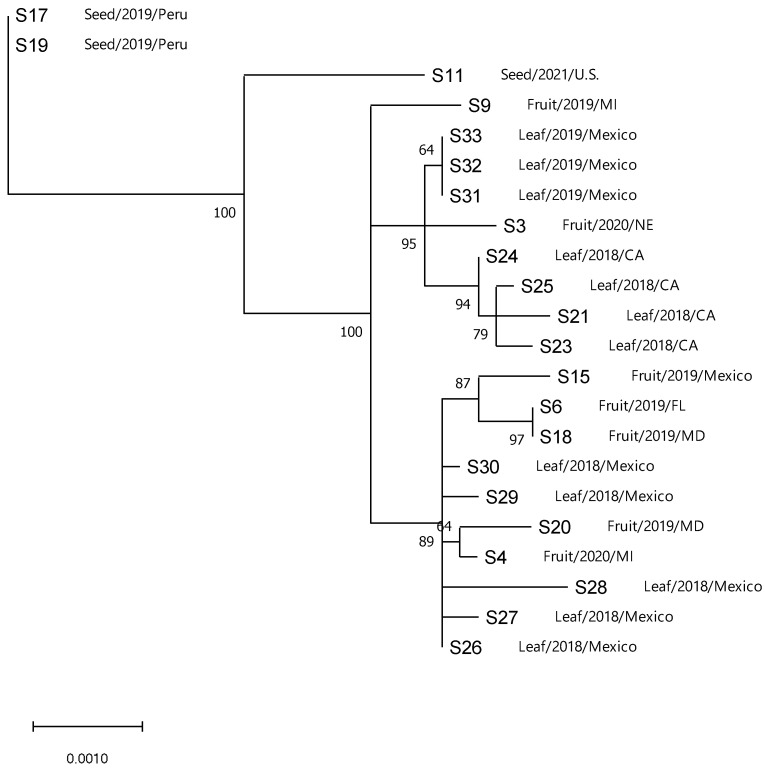
Un-rooted maximum-likelihood tree of tomato brown rugose fruit virus isolates sequenced in this study constructed in PhyML. Description of isolate source, year of isolation, and collection location is indicated following isolate name. Numbers on the branches indicated bootstrap support percentages. The tree was subject to 500 bootstraps. Distance scale represents the number of substitutions per site. Abbreviations: CA: California; FL: Florida; MD: Maryland; MI: Michigan; NE: Nebraska; US: United States.

Phylogenetic analysis of the sequenced isolates, which include local, foreign, and intercepted (*n* = 22), for ToBRFV genomes without the UTR ends showed similar branching and structure (Figure 2). The maximum-likelihood tree showed that most isolates grouped into one clade with high bootstrap support (>90) (Figure 2). Furthermore, isolates showed structure with respect to sample type (i.e., seed, leaf, or fruit). Isolates (S11, S17, S19) from seeds were distantly related to the other isolates collected from leaves or fruits with strong bootstrap support (Figure 2). All other isolates branched in three subclades, one clade containing only one isolate from seed and two others containing isolates from fruits and leaves and from local (U.S.) and foreign (Mexico) localities.

**Figure 3:**
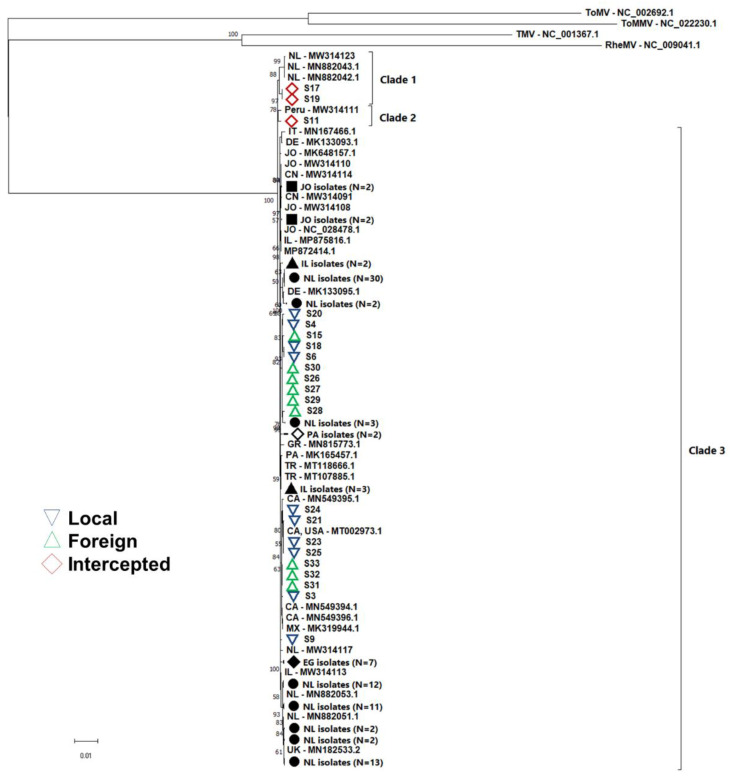
Maximum-likelihood tree of the complete genome sequence for all tomato brown rugose fruit virus (ToBRFV) isolates publicly available and sequenced in this study constructed in PhyML. Closely related tobamovirsues, such as tomato mosaic virus (ToMV), tobacco mosaic virus (TMV), tomato mottle mosaic virus (ToMMV), and Rehmannia mosaic virus (RheMV), were added as outgroups. Branch tips indicate the location of isolation for each isolate, followed by the GenBank accession number. Isolates clustering together from the same geographic region collapsed. Numbers on the branches indicate bootstrap support of more than 50%. Abbreviations: CA: Canada; CA, USA: California, United States of America; CN: China; DE: Germany; EG: Egypt; GR: Greece; IL: Israel; IT: Italy; JO: Jordan; MX: Mexico; NL: Netherlands; PA: Palestinian Authority; TR: Turkey; UK: United Kingdom. The tree was subject to 500 bootstraps. Distance scale represents the number of substitutions per site.

The sequenced isolates (*n* = 22) were combined with publicly available ToBRFV genomes (*n* = 123) for phylogenetic comparison. Furthermore, four closely related tobamoviruses, TMV, ToMV, ToMMV, and Rehmannia mosaic virus (RheMV), were added as outgroups for enhanced tree topology. ToBRFV formed a monophyletic clade with 100% bootstrap support, with the other four tobamoviruses forming separate clades. Interestingly, a similar branching pattern was observed in the phylogenetic tree, which included all ToBRFV sequences compared to the sequences of the U.S. isolates alone, forming three main clades (Figure 2 and Figure 3). Two clades had high bootstrap support above 70, whereas clade 3 had lower bootstrap support at 69 (Figure 3). Clades 1 and 2 contained five and two isolates, respectively. Clade 2 contained isolates from seeds S17 and S19, clustering with Dutch isolates, whereas clade 1 contained one isolate from seed S11 and a Peruvian isolate. Clade 3 contained 138 out of 145 ToBRFV isolates, and several subclades were observed loosely based on geographical location. The multiple sub-clades observed within Clade 3 contained isolates from different geographical areas with low bootstrap support, indicating that most isolates have highly similar sequences and that not enough resolution exists for strong clade formation. Isolates from the U.S. and Mexico groups formed three subclades within Clade 3. The first subclade contained isolates from U.S., Mexico, and the Netherlands, whereas the second subclade contained isolates from the U.S., Mexico, and Canada; both of these subclades had strong bootstrap support. The third subclade contained one isolate from the U.S. and grouped with isolates from Egypt and the Netherlands but with no bootstrap support for this subclade.

### 3.5. Selection Pressure, Population Expansion

The full-length genome and four ORFs of the ToBRFV isolates sequenced in this study (*n* = 22) and all other isolates (*n* = 123) were used to determine various genetic diversity parameters. The *CP* gene had the lowest haplotype diversity (0.476) and nucleotide diversity (0.00112) among all isolates, which was also observed for the isolates sequenced in this study (Table 4). On the other hand, the *MP* gene had the highest nucleotide diversity (0.00362). The RdRp ORFs 183 kDa and 126 kDa had the highest haplotype diversity. Tajima’s D parameter of neutrality for all four genes and the full-length genome sequences showed significant (*p* < 0.05) divergence from neutrality for all isolates combined. Furthermore, Fu and Li’s D * and F * were also significant, confirming the divergence from neutral evolution, indicating population expansion. However, the isolates sequenced in this study showed negative values for Tajima’s D, Fu and Li’s D * and F * but were not significant. The dN/dS ratio was <1 for all ToBRFV genes, except for the *CP* in the isolates sequenced in this study. However, negative selection pressure was observed in the *CP* gene when all isolates were examined. Furthermore, dN/dS ratio was <1 across the whole ToBRFV genome for all isolates combined, indicating strong negative selection pressure in the population (Table 4).

### 3.6. Gene Flow and Genetic Differentiation

Fst and Nm were greater than 0.33 and lower than 1, respectively, when isolates were assigned into sub-populations according to the ToBRFV clades in the phylogenetic tree (Figure 3). Gene flow was not present between these isolates across the three clades, and all showed an Fst > 0.33, indicating that these sub-populations do not share alleles frequently. Furthermore, the low gene flow between the three clades indicates possible early divergence of the seven isolates in clades 1 and 2 (Figure 3, Table 5).

Furthermore, genetic differentiation between the sub-populations was inferred based on different nucleotide-based test statistics, Kst *, Z *, and Snn, which all showed significant *p*-values indicating a genetic distinction between the clades. Inter-population comparisons, such as clade 2 vs. 3 and clade 1 vs. 3, showed strong genetic differentiation. On the other hand, clades 1 and 2 showed lower genetic differentiation from each other. When clades 1 and 2 were combined into one population and compared to clade 3, strong genetic differentiation was confirmed.

## 4. Discussion

In the past decade, a significant increase of viruses, up to 312, was reported to infect tomatoes [36]. ToBRFV was simultaneously reported more than five years ago from Jordan and Israel [1,2], and it continues to pose an imminent threat to tomato production. The virus is known to be seed-borne and has similar properties to other tobamoviruses. Thus, the movement of contaminated or infected plant materials can result in the introduction of ToBRFV to new areas. In order to safeguard agriculture and diagnostic security against new isolates or potential variants that can threaten solanaceous commodities, we characterized the genetic diversity of ToBRFV isolates introduced into the U.S. and worldwide. Several ToBRFV isolates were collected from fruits at retail stores in different states. Moreover, isolates were included from previous surveys in California, prior to ToBRFV eradication, and Mexico. We also analyzed the genetic composition of the genomes and conducted various genetic analyses to understand the drivers of ToBRFV diversity. Based on the results of this study, we found that ToBRFV has very limited genetic diversity across more than 150 genomes. Furthermore, ToBRFV diverges from the neutral evolutionary theory, which indicates the virus is not undergoing natural selection and that accumulated mutations across the genomes are low-frequency and random. Furthermore, this divergence from neutrality is most like caused by a population (geographic) expansion of ToBRFV and is supported by the absence of any structuring in the phylogenetic tree, a high number of haplotypes across different genes, and low nucleotide diversity. These observations indicate that future introductions of ToBRFV should not hamper the integrity of diagnostic tools currently used.

Across the ToBRFV genome, the *CP* gene accumulated the least number of mutations and showed high conservation, as evident by the lowest haplotype diversity and nucleotide diversity among all genes. The reason behind this highly conserved sequence is unknown, but studies in TMV showed that the *CP* gene is highly conserved to preserve elicitor recognition by the *N’* gene in tobacco [37]. Whether this applies to ToBRFV CP requires further examination. On the other hand, the highest nucleotide diversity was observed for the MP gene. Recent studies showed the ToBRFV MP as an essential protein for overcoming resistance in plants containing the *Tm-2^2^* gene [8]. Yan et al. [10] mapped seven amino acids in the central region of the gene critical for ToBRFV overcoming Tm-2^2^ resistance, and the abolishment of any one amino acid rendered the virus non-pathogenic. Several non-synonymous mutations were seen in the MP gene; nevertheless, none were critical amino acid changes, except for one isolate that had a single amino acid change collected from fruit and sequenced in this study. The MP appears to be under negative selection pressure, as any mutations in the critical amino acid positions are lethal to the virus. The majority of amino acid changes in the MP gene occurred in the C-terminus region, which does not affect ToBRFV overcoming Tm-2^2^ resistance. Furthermore, all genes showed negative or purifying selection pressure pointing to an undergoing population expansion based on different genetic parameters. Negative selection pressure has been commonly found in other tobamoviruses, such as tobacco mosaic virus, tobacco mild green mosaic virus, tomato mosaic virus (ToMV), and pepper mild mottle virus [38,39,40]. For instance, the MP gene, which is the avirulence gene of ToMV that interacts with the *Tm-2^2^* resistance gene, has a dN/dS ratio of 0.314 [41]. Tobamoviruses exhibit strong negative selection in avirulence (*Avr*) genes (e.g., *CP*) interacting with *R* genes [40]. It is likely that the negative selection observed in the ToBRFV genome is similar to those observed in Avr-R gene interactions in other tobamoviruses. The ToBRFV isolates sequenced in this study had a slightly higher number of non-synonymous substitutions across all four genes compared to the Dutch isolates sequenced by van de Vossenberg et al. [24]. For instance, the Dutch isolates from 2020 had up to six non-synonymous substitutions in the RdRp gene compared to eight substitutions in our sequenced isolates. The MP gene in the Dutch isolates had five mutations compared to six in our study, whereas the *CP* gene did not have non-synonymous substitutions. Furthermore, the higher number of non-synonymous to synonymous mutations observed in the *CP* gene sequences of our sequenced isolates is likely related to the sample size. Janzac et al. [41] have shown that increasing the sequence sample size can affect the dN/dS ratio.

The first ToBRFV outbreak was reported only six years ago, and the virus is still undergoing geographical expansion across the globe. In the past three years, reports of ToBRFV infection have increased in the literature. Growers and stakeholders are rapidly eradicating the virus, which can lead to a genetic bottleneck which we have seen reflected in the genetic data in this study. Genome sequencing was harnessed to track and monitor ToBRFV virus outbreaks in the Netherlands [29]. Eradication efforts were monitored by sequencing isolates before and after control measures to assess proper disease management [29]. Phylogenetic analysis of all isolates revealed three clades for the ToBRFV populations with strong genetic differentiation. The phylogenetic tree indicates early divergence of some of the ToBRFV isolates, such as those in clades 1 and 2. Seven isolates across clades 1 and 2, three of which were collected in this study, appear to diverge from the majority of isolates in clade 3. Interestingly four of the seven isolates across clades 1 and 2 were isolated from seeds indicating potential sources of diversity in seed-borne ToBRFV. Our analyses support the hypothesis put forth by van de Vossenberg et al. [29] with regard to the divergence of the isolates from Peru, suggesting that ToBRFV potentially originated from South America. However, more isolates from South America and other seed-producing areas are needed to confirm this hypothesis. Furthermore, globalization and seed trade make it difficult to identify a single source for ToBRFV outbreaks. For instance, ToBRFV has been reported in the Netherlands from imported seeds from Peru, China, Israel, and Jordan [42,43]. On the other hand, the majority of isolates that clustered within clade 3 showed high gene flow and a lack of proper structure even across geographical areas, which was supported by the low bootstrap support across different branches of the phylogeny. As a result, we conclude that ToBRFV isolates from all geographic regions show a high level of interrelatedness, low genetic diversity, and random mutations across genomes. This can be explained by the fact that most ToBRFV outbreaks are attributed to the introduction of infected seeds and the simultaneous introduction of ToBRFV into multiple countries.

## 5. Conclusions

In this study, we have identified a limited number of isolates with higher diversity than the majority of the ToBRFV isolates in the population. Further studies are needed to evaluate more isolates from various geographical regions, especially seed-producing areas. The limited diversity and random mutations across various ToBRFV sequences indicate that diagnostic assays should remain effective in detecting ToBRFV. However, periodic surveys to monitor ToBRFV diversity are essential to understanding potential population shifts in the future and avoiding threats from recombinant strains that can render diagnostic assays ineffective.

## Figures and Tables

**Table 1:** Tomato brown rugose fruit virus (ToBRFV) isolates sequenced in this study.

Isolate	Plant Material	Date Received	Source	Location ^a^	Real-Time PCR (Ct) ^b^
S3	Fruit	April 2020	Retail store	Nebraska, U.S.	7.9
S4	Fruit	February 2020	Retail store	Michigan, U.S.	6.7
S6	Fruit	November 2019	Retail store	Florida, U.S.	9.4
S9	Fruit	December 2019	Retail store	Michigan, U.S.	11.1
S11	Seed	March 2021	Seed producer	U.S. ^c^	9.8
S15	Fruit	December 2019	Import	Mexico	6.5
S17	Seed	August 2019	Seed producer	Peru	16.5
S18	Fruit	December 2019	Retail store	Maryland, U.S.	NT ^d^
S19	Seed	August 2019	Seed producer	Peru	9.5
S20	Fruit	December 2019	Retail store	Maryland, U.S.	8.7
S21	Leaf	November 2018	Greenhouse	California, U.S.	NT
S23	Leaf	November 2018	Greenhouse	California, U.S.	24.82
S24	Leaf	November 2018	Greenhouse	California, U.S.	21.12
S25	Leaf	November 2018	Greenhouse	California, U.S.	NT
S26	Leaf	November 2018	Greenhouse	Mexico	6.76
S27	Leaf	November 2018	Greenhouse	Mexico	8.26
S28	Leaf	November 2018	Greenhouse	Mexico	6.35
S29	Leaf	November 2018	Greenhouse	Mexico	6.26
S30	Leaf	November 2018	Greenhouse	Mexico	16.38
S31	Leaf	February 2019	Greenhouse	Mexico	14.02
S32	Leaf	February 2019	Greenhouse	Mexico	11.91
S33	Leaf	February 2019	Greenhouse	Mexico	8.14

^a^ Samples collected from fruits at U.S. retail stores are of an unknown production origin. Other samples were collected at production greenhouses or imported from the source of origin. ^b^ RT-qPCR: reverse transcription real-time PCR; based on ToBRFV MP gene [3]. ^c^ Origin is foreign but intercepted in U.S. ^d^ Not tested.

**Table 2:** Tomato brown rugose fruit virus high-throughput sequencing data generated for the isolates in this study.

Isolate	Raw Reads	No. Mapped Reads ^a^	Mean Coverage	% RefSeq ^b^	Draft Genome Size (bp)
S3	13,462,663	3,809,241	42,288	99.8	6376
S4	14,057,467	9,734,997	117,165	99.9	6380
S6	15,273,550	11,035,573	125,255	99.9	6380
S9	13.042,460	2,771,812	31,097	99.9	6380
S11	50,476,803	2,386,377	26,954	99.9	6382
S15	12,246,915	8,521,243	96,252	99.9	6381
S17	66,390,808	3,177,034	35,528	99.9	6381
S18	63,394,513	51,801,176	715,817	99.9	6392
S19	78,158,737	65,509,729	773,145	99.9	6393
S20	226,676,066	48,291,439	604,169	99.6	6375
S21	47,616,520	46,470,185	598,411	99.9	6384
S23	32,736,111	6701	78	99.4	6357
S24	33,270,742	8448	99	99.6	6371
S25	20,186,935	8,722,599	104,912	99.9	6392
S26	26,239,529	23,870,654	313,051	99.9	6384
S27	35,553,785	16,118,484	202,182	99.9	6380
S28	19,230,903	17,252,098	218,573	99.8	6383
S29	12,671,194	8,202,718	101,900	99.8	6383
S30	39,047,819	14,943,377	184,625	100.0	6393
S31	35,645,442	31,696,564	408,411	99.9	6384
S32	48,390,993	35,607,924	448,364	99.9	6386
S33	30,027,166	26,643,010	342,051	99.8	6384

^a^ Number of reads with a Phred score of Q30. ^b^ ToBRFV GenBank accession no. NC_028478.1 was used as a reference.

**Table 3:** Tomato brown rugose fruit virus genome statistics of sequenced isolates in this study.

Gene	Sequence Length (bp)	Nt Identity ID % ^a^	A.A. Identity % ^b^	Synonymous Mutations	Non-Synonymous Mutation
183 kDa	4848	99.78	99.94	53	8
126 kDa	3351	99.78	99.96	36	4
MP	801	99.63	99.41	10	6
CP	480	99.88	99.71	1	4

^a^ Nt: Nucleotide. ^b^ A.A.: Amino acid.

**Table 4:** Genetic diversity parameters for tomato brown rugose fruit virus genomes sequenced in this study (*n* = 22) and combined with publicly available genomes (*n* = 145).

Gene	Sequence Length (bp)	N ^a^	H ^b^	*S* ^c^	π ^d^	Hd ^e^	Tajima’s D ^f^	Fu and Li’s D * ^g^	Fu and Li’s F * ^h^	dN/dS ^i^
RdRp (183 kDa)	4848	22	17	60	0.00220	0.974	−1.44271	−1.55880	−1.78233	0.029
		144 ^j^	85	220	0.00238	0.987	−2.32187 **	−3.83944 **	−3.75501 **	0.094
RdRp (126 kDa)	3351	22	16	40	0.00219	0.965	−1.29515	−1.41931	−1.61486	0.019
		144 ^j^	78	149	0.00221	0.981	−2.34105 **	−3.84386 **	−3.78484 **	0.079
MP	801	22	11	17	0.00362	0.892	−1.38241	−1.30950	−1.55103	0.377
		145	40	48	0.00414	0.923	−1.93509 *	−3.47504 **	−3.38850 **	0.192
CP	480	22	6	5	0.00112	0.476	−1.80901 *	−2.09702	−2.33018	1.525
		145	22	25	0.00190	0.644	−2.27816 **	−3.92019 **	−3.92314 **	0.116
Full-length	6117	22	18	82	0.00230	0.978	−1.52835	−1.66511	−1.90015	0.123 ^k^
		145	96	294	0.00257	0.992	−2.31476 **	−4.15842 **	−3.94355 **	0.413 ^k^

^a^ Number of isolates analyzed, the 1st row indicates the isolates sequenced in this study, and the 2nd row indicates number of isolated sequenced in this study combined with publicly available genomes. ^b^ Number of haplotypes. ^c^ Number of segregating (polymorphic) sites. ^d^ Nucleotide diversity. ^e^ Gene diversity. ^f^ Indicates *: *p* < 0.05; **: *p* < 0.01. ^g^ Indicates *: *p* < 0.05; **: *p* < 0.02. ^h^ Indicates *: *p* < 0.05; **: *p* < 0.02. ^i^ ratio of the number of nonsynonymous substitutions to the number of synonymous substitutions. ^j^ ToBRFV isolate Palestinian (GenBank accession no. MK165457.1) was excluded. ^k^ In coding region only.

**Table 5:** Inter-population diversity of tomato brown rugose fruit virus.

Population	Fst ^a^	Nm ^b^	Kst *	Z *	Snn
Clade 1 vs. 2	0.42279	0.34	0.21000 * ^c^	1.67244 *	1.0 ns^d^
Clade 1 vs. 3	0.53847	0.21	0.02486 ***	8.14475 ***	1.0 ***
Clade 2 vs. 3	0.36199	0.44	0.00671 ***	8.16035 ***	1.0 ***
Clade 1 and 2 vs. 3	0.40248	0.37	0.02507 ***	8.16805 ***	1.0 ***

^a^ Fixation index. ^b^ Number of migrants per generation. ^c^ Indicates *: *p* < 0.05; ***: *p* < 0.001. ^d^ ns: non-significant.

## Data Availability

The raw output sequencing files and genome sequences are available upon reasonable request.
